# Twenty-Two Years of Prenatal Testing for Suspected Monogenic Disorders: A Retrospective Single-Center Experience in Western Romania

**DOI:** 10.3390/medsci14050548

**Published:** 2026-09-07

**Authors:** Miruna Gug, Nicoleta Andreescu, Eugen Dan Chicea, Adrian Rațiu, Simona Farcaș, Ioana Marin, Stelian Țîcău, Cristina Gug

**Affiliations:** 1 Doctoral School of Medicine, “Victor Babes” University of Medicine and Pharmacy, 300041 Timisoara, Romania; miruna.gug@umft.ro; 2 Discipline of Medical Genetics, Department of Microscopic Morphology, Faculty of Medicine, “Victor Babeș” University of Medicine and Pharmacy, 300041 Timișoara, Romania; farcas.simona@umft.ro (S.F.); cristina.gug@umft.ro (C.G.); 3 Genomic Medicine Centre, “Victor Babeș” University of Medicine and Pharmacy, 300041 Timișoara, Romania; 4 Regional Centre of Medical Genetics Timiș (CRGMT), “Louis Țurcanu” Clinical Emergency Hospital for Children, 300011 Timișoara, Romania; 5 Medical Genetics Office Doctor Gug, 300200 Timișoara, Romania; 6 Master’s Program in Bioinformatics, Faculty of Informatics, West University of Timișoara, 300223 Timișoara, Romania; ticaustelian@gmail.com; 7 Interdisciplinary Doctoral School, Transilvania University of Brașov, 500019 Brașov, Romania; 8 Clinical County Emergency Hospital Sibiu, 550245 Sibiu, Romania; 9 Department of Obstetrics and Gynecology II, “Victor Babeș” University of Medicine and Pharmacy, 300041 Timișoara, Romania; ratiu.adrian@umft.ro; 10 Timișoara Municipal Emergency Clinical Hospital, 300202 Timișoara, Romania; 11 Department of Microbiology, Discipline of Hygiene, Center for Studies in Preventive Medicine, “Victor Babeș” University of Medicine and Pharmacy, 300041 Timișoara, Romania; ioana.marin@umft.ro

**Keywords:** prenatal diagnosis, monogenic disorders, prenatal genomics, whole-exome sequencing, cell-free DNA, genetic counseling, medical genetics, Romania

## Abstract

Background/Objectives: Prenatal testing strategies for suspected monogenic disorders have changed considerably over the past decades, alongside evolving referral indications and increasing availability of genomic technologies. Longitudinal, practice-based data describing these changes within routine clinical care remain limited, particularly in Central and Eastern Europe. We describe changes in referral indications, testing strategies, and molecular findings over 22 years in a single-center retrospective descriptive case series from Western Romania. Methods: We conducted a retrospective analysis of 52 pregnancies investigated for suspected monogenic disorders between 2004 and 2026. Forty pregnancies were evaluated through a diagnostic pathway, while a separate group of 12 pregnancies underwent cell-free DNA (cfDNA)-based monogenic screening; these were analyzed as distinct clinical pathways. For the diagnostic pathway, clinical indications, testing strategies, and molecular findings were analyzed across three retrospectively defined study periods (2004–2013, 2014–2019, and 2020–2026). Results: Within the diagnostic pathway, referral patterns shifted from predominantly family-history-based testing (55.6% of cases in 2004–2013) toward indications arising from positive parental carrier screening and fetal ultrasound abnormalities in later study periods. Testing strategies expanded from predominantly targeted single-gene testing and multiplex ligation-dependent probe amplification (MLPA) to include gene panels and whole-exome sequencing (WES). Among 34 pregnancies with fetal molecular evaluation, 7 (20.6%) had a confirmed disease-causing finding, 9 (26.5%) had carrier-only outcomes, 15 (44.1%) were classified as unaffected, and 3 (8.8%) had non-classic molecular findings. Incidental or additional molecular findings beyond the primary testing indication were identified in 6 of 34 pregnancies with fetal molecular evaluation (17.6%) and required case-specific interpretation and genetic counseling. The 12 cfDNA-based monogenic screening pregnancies constituted a separate, non-diagnostic screening pathway and were analyzed independently from the diagnostic pathway. Conclusions: Over 22 years, referral indications for suspected monogenic disorders broadened alongside an expansion of prenatal testing strategies from predominantly targeted familial testing to a wider range of genomic approaches. These findings describe temporal changes within a single-center clinical practice and should not be interpreted as evidence that changes in testing strategy improved diagnostic performance or pregnancy outcomes. Invasive diagnostic testing and cfDNA-based monogenic screening represent distinct clinical pathways and should be interpreted separately.

## 1. Introduction

Prenatal molecular diagnosis has changed over the past two decades, evolving from single-gene testing targeted at a known familial variant toward genome-wide and, more recently, cell-free DNA (cfDNA)-based approaches [1,2]. Test selection is no longer determined by a single default technology. Expanded carrier screening identifies couples at increased reproductive risk and creates a distinct indication for targeted fetal testing [3,4]. An abnormal fetal ultrasound finding may instead prompt exome sequencing when microarray chromosomal testing is non-diagnostic, and cfDNA-based screening offers a separate screening route that still requires confirmatory invasive testing when positive [5]. Referred pregnancies therefore no longer share a single clinical profile, and the appropriate strategy depends on the specific context of each pregnancy.

Most prenatal exome studies have focused on fetuses with structural anomalies [6,7]. Few studies have examined how prenatal molecular diagnostic strategies have evolved over time within routine clinical practice, integrating changing referral indications, technological change, and genetic counseling within a single, continuously followed cohort. Longitudinal, practice-based evidence of this kind is needed to understand how genomic technologies have actually been adopted into everyday prenatal genetic practice alongside targeted testing.

Clinical genetics capacity and access to genomic testing remain uneven across Europe, with limited longitudinal data from Central and Eastern European settings [8,9]. Romanian data on the clinical management of pregnancies following NIPT were first reported in 2022 and subsequently expanded [10,11]. National data on carrier frequencies and couple-based reproductive carrier screening have also begun to emerge [12,13], although practice-based data describing how prenatal molecular diagnosis has evolved in this region remain limited. Similar efforts to expand preconception and reproductive carrier screening have recently been reported in other Central and Eastern European settings, including a pilot carrier-screening program in Russia [14] and a structured preconception screening pathway in Austria [15].

This study was designed as a retrospective longitudinal descriptive case series of pregnancies investigated for suspected monogenic disorders within a single private referral practice in Western Romania. The primary objective was to describe how referral indications, testing strategies, and molecular findings changed over time among pregnancies evaluated within this practice between 2004 and 2026. The study was not designed to evaluate whether individual testing strategies improved clinical outcomes. It was restricted to pregnancies investigated for a monogenic indication; routine prenatal cytogenetic and chromosomal testing formed a separate component of clinical activity and was not included. We describe a cohort of 52 pregnancies included in two conceptually and analytically distinct clinical pathways: a diagnostic pathway (*n* = 40) and a cfDNA-based monogenic screening pathway (*n* = 12). We report changes in referral indications, testing strategies, and molecular findings across three study periods, together with genetic counseling considerations observed in this setting. As a single-center descriptive study without a defined catchment population or comparator group, this analysis cannot establish causal or comparative effects of individual testing strategies or support population-level inferences.

## 2. Materials and Methods

### 2.1. Study Design and Population

This retrospective longitudinal descriptive case series evaluated 52 pregnancies investigated for suspected monogenic disorders between 2004 and 2026 at a single private outpatient medical genetics clinic in Western Romania (Timișoara). The study describes temporal changes in clinical practice within this selected referral population. It does not define a catchment population, a denominator of all pregnancies or referrals seen at the clinic, or a population-based ascertainment framework and therefore cannot support inferences regarding prevalence, incidence, or population-level uptake of prenatal monogenic testing. Cases were identified from the departmental clinical database, which recorded 58 records over the study period; 6 records were excluded from the analytic cohort (3 maternal-only investigations without fetal molecular testing, 1 preconceptional investigation, and 2 postnatal index cases retained only to document familial molecular context; Section 3.1). Records limited to fetal karyotyping for aneuploidy, biochemical screening unrelated to a monogenic indication, or non-invasive fetal sex determination performed in the absence of a monogenic risk were not eligible for inclusion in the analytic cohort. The final cohort comprised two conceptually and analytically distinct clinical pathways: a diagnostic pathway (*n* = 40), which included invasive fetal sampling and/or fetal-sex determination for X-linked risk (including two pregnancies resolved by non-invasive fetal-sex determination alone), and a cfDNA-based monogenic screening pathway (*n* = 12; available from 2024 onward). These pathways were analyzed separately because of their distinct clinical indications, purposes, and denominators. Data were extracted and anonymized from clinical and laboratory records, including maternal age, gestational age at sampling, clinical indication, diagnostic strategy, molecular results, and pregnancy or neonatal outcome where available. As a retrospective analysis of anonymized clinical data collected during routine care, formal Institutional Review Board approval and the applicable consent procedures are detailed in the Institutional Review Board Statement and Informed Consent Statement sections of this manuscript.

Routine prenatal cytogenetic and chromosomal investigations, including conventional karyotyping, fluorescence in situ hybridization (FISH), chromosomal microarray analysis (CMA), and chromosomal non-invasive prenatal testing (NIPT) for common aneuploidies or other chromosomal abnormalities, constituted a separate component of the clinic’s activity and were not included in this analysis, unless karyotyping or fetal sex determination formed part of the diagnostic pathway for a suspected monogenic or X-linked condition (Section 3.1).

### 2.2. Clinical Evaluation and Prenatal Diagnostic Workflow

All pregnancies underwent pre-test genetic counseling, during which the clinical indication for testing was established, family history was reviewed across at least three generations, and additional family members were offered testing, where relevant, to clarify the segregation of a suspected pathogenic variant. Clinical indications within the diagnostic pathway included a previously affected child or family member, familial X-linked risk, positive parental carrier screening, fetal ultrasound abnormalities, and an affected parent with a known autosomal dominant condition (Section 3.2). Referral for cfDNA-based monogenic screening was driven predominantly by advanced parental age, especially paternal age, and routine screening in the absence of a specific monogenic suspicion.

Invasive sampling consisted of chorionic villus sampling (10–13 weeks) or amniocentesis (from 15 weeks), performed under continuous ultrasound guidance by an experienced operator. The diagnostic strategy, whether targeted familial variant testing, a gene panel, whole-exome sequencing, or, when appropriate, non-invasive fetal sex determination as a first step in X-linked conditions, was selected based on the known familial variant (when available), the fetal phenotype, and the range of technologies accessible to the practice at the time of testing; this selection was not governed by a predefined algorithm (Section 3.3). All results were disclosed during post-test genetic counseling, which included recurrence-risk assessment and discussion of the implications for the current and future pregnancies (Section 3.5).

For descriptive temporal analysis, the 22-year study interval was divided retrospectively into three study periods (2004–2013, 2014–2019, and 2020–2026), based on changes in the range of molecular testing technologies available to and used within this clinical practice. These periods were defined as analytical categories for the present study and should not be interpreted as predefined clinical or technological eras.

### 2.3. Molecular Genetic Testing

Within the diagnostic pathway, molecular testing strategies used over the study period included targeted mutation testing by polymerase chain reaction (PCR), multiplex ligation-dependent probe amplification (MLPA, primarily for *DMD* analysis but also for *SMN1* deletion/duplication analysis), Sanger sequencing for known familial variants, NGS-based testing, and whole-exome sequencing (WES), alongside non-invasive or karyotype-based fetal sex determination when appropriate for X-linked risk. The cfDNA-based monogenic screening pathway was analyzed separately, as described below. Targeted *CFTR* mutation testing performed in 2008 used the Elucigene CF29 assay (Tepnel Diagnostics Ltd., Abingdon, UK), based on allele-specific amplification (ARMS-PCR), which screened for 29 *CFTR* mutations. Testing was performed at national public laboratories, Romanian private laboratories, and international reference laboratories, depending on the strategy, period, and specific gene or panel required. Laboratories used for testing changed over the study period, from national public laboratories (e.g., for MLPA-based analyses) to Romanian private laboratories and international reference laboratories offering gene-panel and whole-exome sequencing, reflecting the technologies available to the practice at each point in time rather than a planned migration strategy. Platforms, gene content, and bioinformatic pipelines for panel- and WES-based testing were defined and validated by each reporting laboratory according to its own protocols; laboratory-specific coverage and quality-control metrics were not systematically available in the retrospective clinical records across the full study period.

Clinically relevant positive findings identified by NGS-based testing (gene panels and WES) were confirmed using an orthogonal method, typically Sanger sequencing for sequence variants or MLPA for copy-number changes, in accordance with the reporting laboratory’s standard validation procedures. Procedures used to minimize or assess maternal cell contamination in invasive prenatal samples changed over the study period. In earlier years, molecular testing was performed on cultured fetal cells, thereby reducing the contribution of maternal cells to the analyzed material. In more recent years, maternal cell contamination has been assessed directly by molecular comparison of fetal and maternal DNA, according to the protocols of the testing laboratory. Because these procedures varied across laboratories and over time, a uniform molecular assessment of maternal cell contamination cannot be documented for the entire retrospective cohort.

cfDNA-based monogenic screening was performed on maternal peripheral blood using commercial targeted NGS assays (NIFTY-MONO, BGI Genomics, Shenzhen, China), with panel content expanding over the study period. From its introduction into the practice in 2024, the assay evolved through three successive panel versions, expanding from 18 genes (2038 pathogenic or likely pathogenic [P/LP] variants) to 155 genes (6246 P/LP variants) and ultimately to 155 genes encompassing more than 22,500 P/LP variants across 218 monogenic disorders. A screen-positive cfDNA result was considered a screening finding requiring confirmatory invasive testing, reflecting the non-diagnostic nature of cfDNA-based monogenic screening.

### 2.4. Variant Interpretation and Statistical Analysis

Sequence variants were classified according to the American College of Medical Genetics and Genomics (ACMG) and Association for Molecular Pathology (AMP) five-tier classification system (pathogenic, likely pathogenic, variant of uncertain significance [VUS], likely benign, benign), reported using Human Genome Variation Society (HGVS) nomenclature and cross-referenced against ClinVar and gene-specific locus databases where applicable. Molecular and clinical data were compiled and analyzed using (Microsoft Excel for Microsoft 365, Version 2608 (Microsoft Corporation, Redmond, WA, USA). Data processing and figure generation were additionally performed using Python, Version 3.12 (Python Software Foundation, Wilmington, DE, USA). Given the descriptive nature and limited cohort size, results are reported as counts and percentages for categorical variables and as means ± standard deviation or medians with ranges for continuous variables, without formal inferential hypothesis testing.

For analysis, molecular findings were categorized according to their relationship to the primary indication for testing. Pathogenic or likely pathogenic findings explaining the suspected fetal disorder were distinguished from carrier findings, variants of uncertain significance, and incidental or additional findings unrelated to the primary indication. Carrier status, VUS, and incidental or additional findings were not considered diagnostic confirmations of the disorder under investigation.

Variants classified as VUS were not considered diagnostic findings and were interpreted in the context of the fetal phenotype, family history, and segregation information where available. Incidental or additional findings identified through broader genomic testing were evaluated individually with respect to their clinical relevance and were addressed during genetic counseling. Because testing approaches and reporting practices changed over the 22-year study period, a uniform retrospective disclosure framework cannot be documented across the entire cohort.

## 3. Results

### 3.1. Study Cohort and Patient Characteristics

Between 2004 and 2026, 58 clinical records were identified in the departmental database as potentially eligible for this study. Six records were excluded from the analytic cohort, as detailed in Section 2.1; none represented a pregnancy undergoing prenatal fetal molecular testing. The final study cohort comprised 52 pregnancies, evaluated through two conceptually and analytically distinct clinical pathways: a diagnostic pathway (*n* = 40) and a cfDNA-based monogenic screening pathway (*n* = 12; available from 2024 onward). The cfDNA pathway was analyzed as a separate, non-diagnostic screening route, with confirmatory invasive diagnostic testing required for screen-positive findings. Individual pregnancy-level characteristics are provided in Supplementary Table S1.

Within the diagnostic pathway, 34 of 40 pregnancies underwent direct fetal molecular testing following invasive fetal sampling. In the remaining six pregnancies, no further fetal molecular testing was undertaken, as non-invasive or karyotype-based fetal sex determination identified a female fetus in each case, excluding the X-linked condition under investigation in that family (*DMD*, *n* = 5; haemophilia A, *n* = 1).

**Figure 1:**
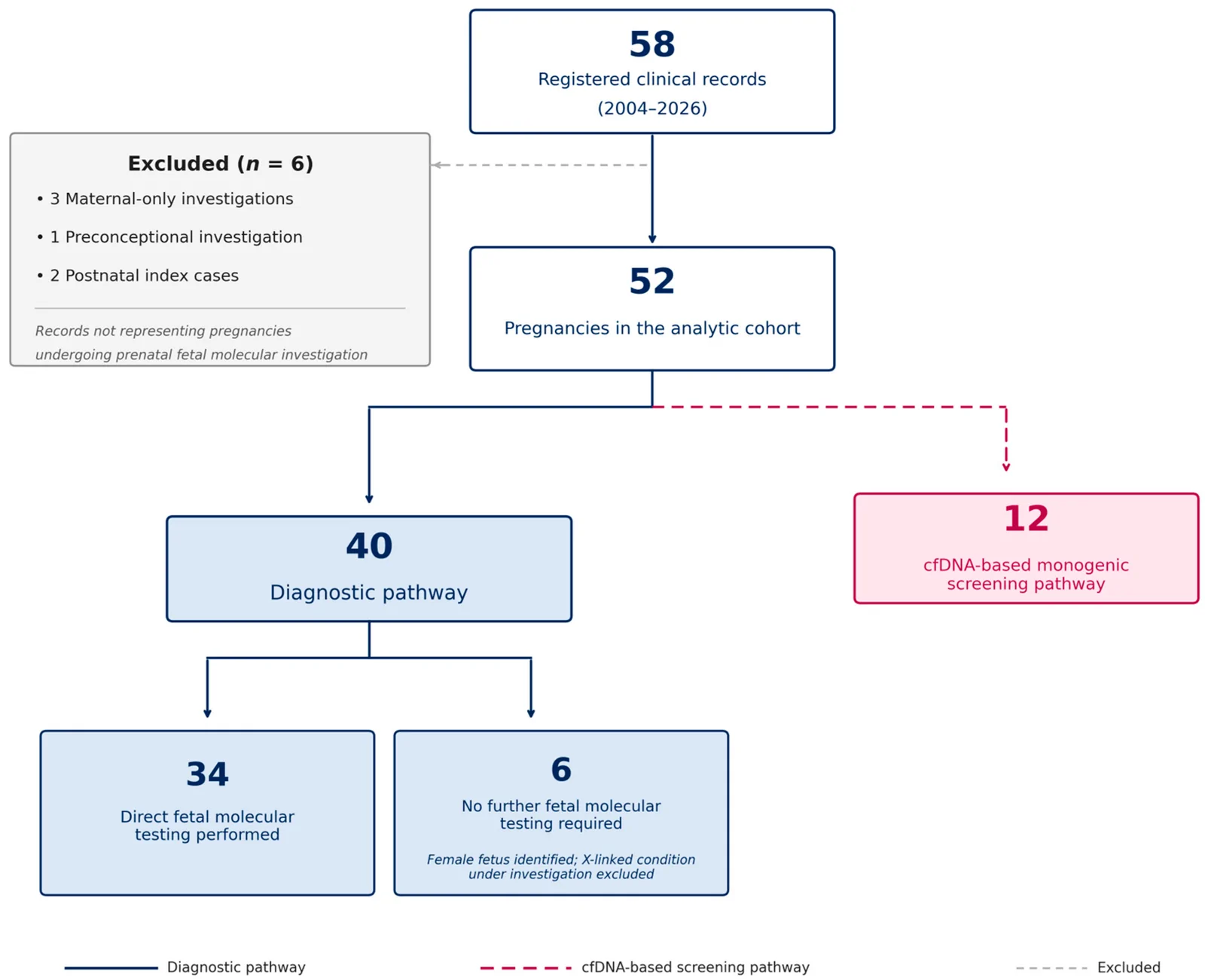
Derivation of the analytic cohort. Of 58 clinical records identified between 2004 and 2026, six were excluded because they did not represent pregnancies undergoing prenatal fetal molecular investigation. The final analytic cohort comprised 52 pregnancies: 40 in the diagnostic pathway and 12 in the cfDNA-based monogenic screening pathway. Within the diagnostic pathway, direct fetal molecular testing was performed in 34 pregnancies; in six pregnancies at risk for X-linked disorders, fetal sex determination identified a female fetus and no further fetal molecular testing was required.

These six pregnancies are reported as “no further testing” rather than as missing data, since fetal sex determination had already resolved the diagnostic question (Figure 1).

Baseline characteristics of the cohort are summarized in Table 1. Maternal age ranged from 19 to 46 years (mean 31.7 ± 5.9 years; median 31 years), with a median age of 38 years in the cfDNA pathway and 30 years in the diagnostic pathway, consistent with the predominance of advanced maternal and paternal age among referral indications for cfDNA screening. Paternal age was systematically available only for pregnancies managed through the cfDNA pathway, where it ranged from 32 to 54 years (mean 41.7 years; median 43 years). Gestational age at sampling ranged from 10 to 24 weeks (median 16 weeks). All pregnancies were singleton. Within the diagnostic pathway, 9 pregnancies were investigated during the first study period (2004–2013), 14 during the second study period (2014–2019), and 17 during the third study period (2020–2026). The cfDNA-based monogenic screening pathway (*n* = 12) became available only during the third study period and is reported separately from the diagnostic pathway throughout the analyses (Section 3.4.2).

### 3.2. Changing Clinical Indications for Prenatal Testing for Suspected Monogenic Disorders

Among the 40 pregnancies investigated through the diagnostic pathway, a previously affected child or family member was the most frequent clinical indication (*n* = 16), followed by positive parental carrier screening and fetal ultrasound abnormalities (*n* = 9 each). Familial X-linked risk accounted for four pregnancies, while a parental molecular diagnosis, defined as an affected parent with an autosomal dominant condition, accounted for two pregnancies. Indications were assigned as a single, dominant category per pregnancy. The cfDNA-based monogenic screening pathway (*n* = 12) followed a distinct referral logic, driven predominantly by advanced maternal or paternal age and routine screening in the absence of a specific monogenic suspicion, and was therefore not included in this indication-based analysis.

**Figure 2:**
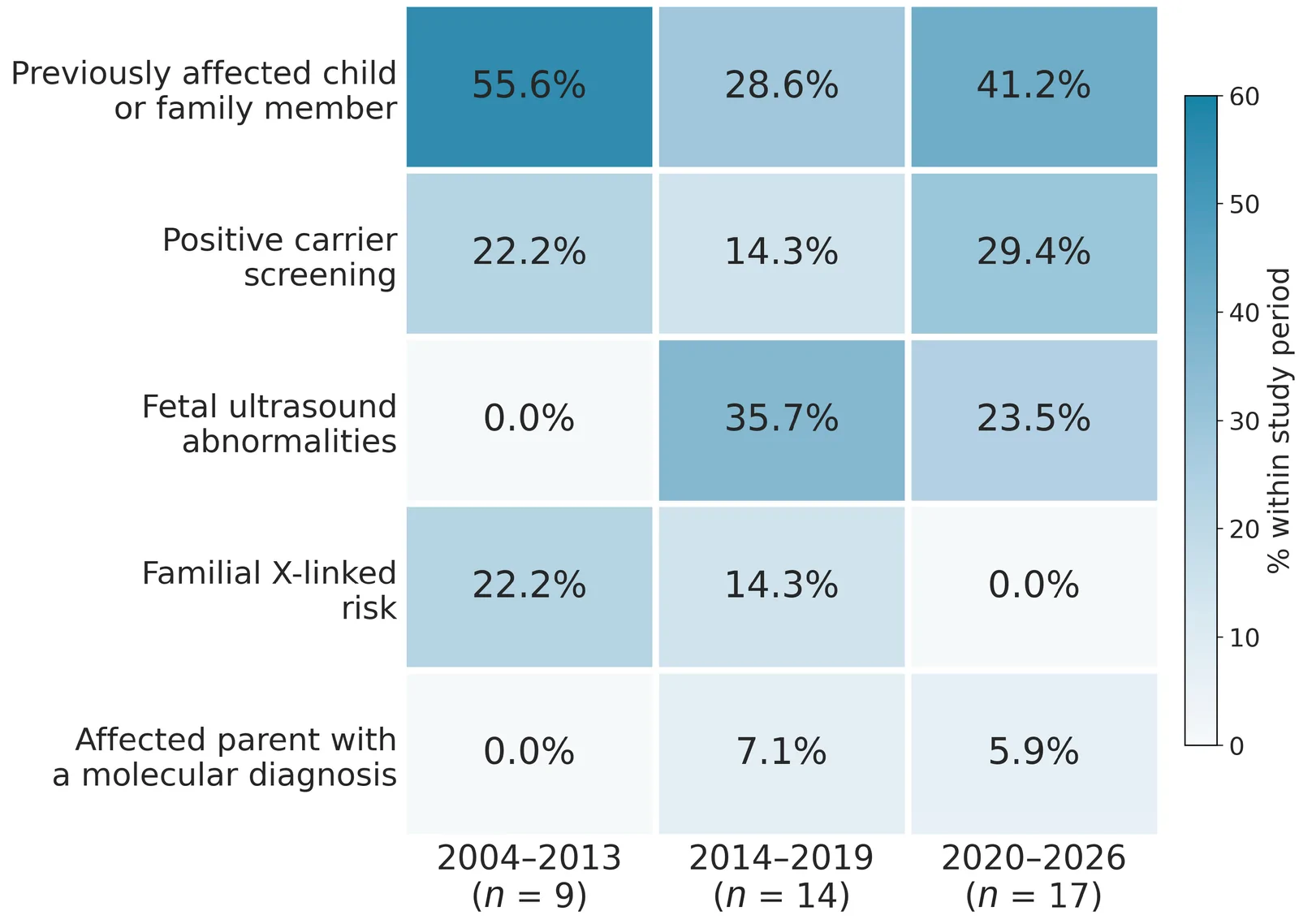
Distribution of primary clinical indications within the diagnostic pathway (*n* = 40) across the three study periods. Values represent the percentage of pregnancies within each study period; the corresponding number of pregnancies is shown below each period.

Referral patterns within the diagnostic pathway changed across the three study periods (Figure 2). In the first study period (2004–2013, *n* = 9), a previously affected child or family member was the dominant indication (55.6%), together with familial X-linked risk and positive parental carrier screening (22.2% each); fetal ultrasound abnormalities were not represented. In the second study period (2014–2019, *n* = 14), fetal ultrasound abnormalities were the leading indication (35.7%), a previously affected child or family member remained frequent (28.6%), and parental molecular diagnosis was first represented (7.1%). In the third study period (2020–2026, *n* = 17), a previously affected child or family member remained the single most frequent indication (41.2%), followed by positive parental carrier screening (29.4%) and fetal ultrasound abnormalities (23.5%), while familial X-linked risk was not represented in this subgroup.

A previously affected child or family member remained the most frequent family-history indication throughout the entire study period. Fetal ultrasound findings and parental molecular diagnosis were first represented as referral indications in this series during the second study period and continued to be represented in the third study period (Figure 2).

### 3.3. Testing Strategies Used Within the Diagnostic Pathway

Within the diagnostic pathway (*n* = 40), seven testing strategies were used: fetal sex determination, targeted *CFTR* mutation testing by PCR, MLPA (deletion/duplication analysis), targeted familial variant testing (Sanger sequencing), single-gene NGS panels, multigene NGS panels, and whole-exome sequencing (WES). These strategies ranged from targeted confirmation or exclusion of a known familial variant to broader panel or exome-based testing. The cfDNA-based monogenic screening pathway (*n* = 12) is reported separately (Section 3.1) and is not included in this analysis.

**Figure 3:**
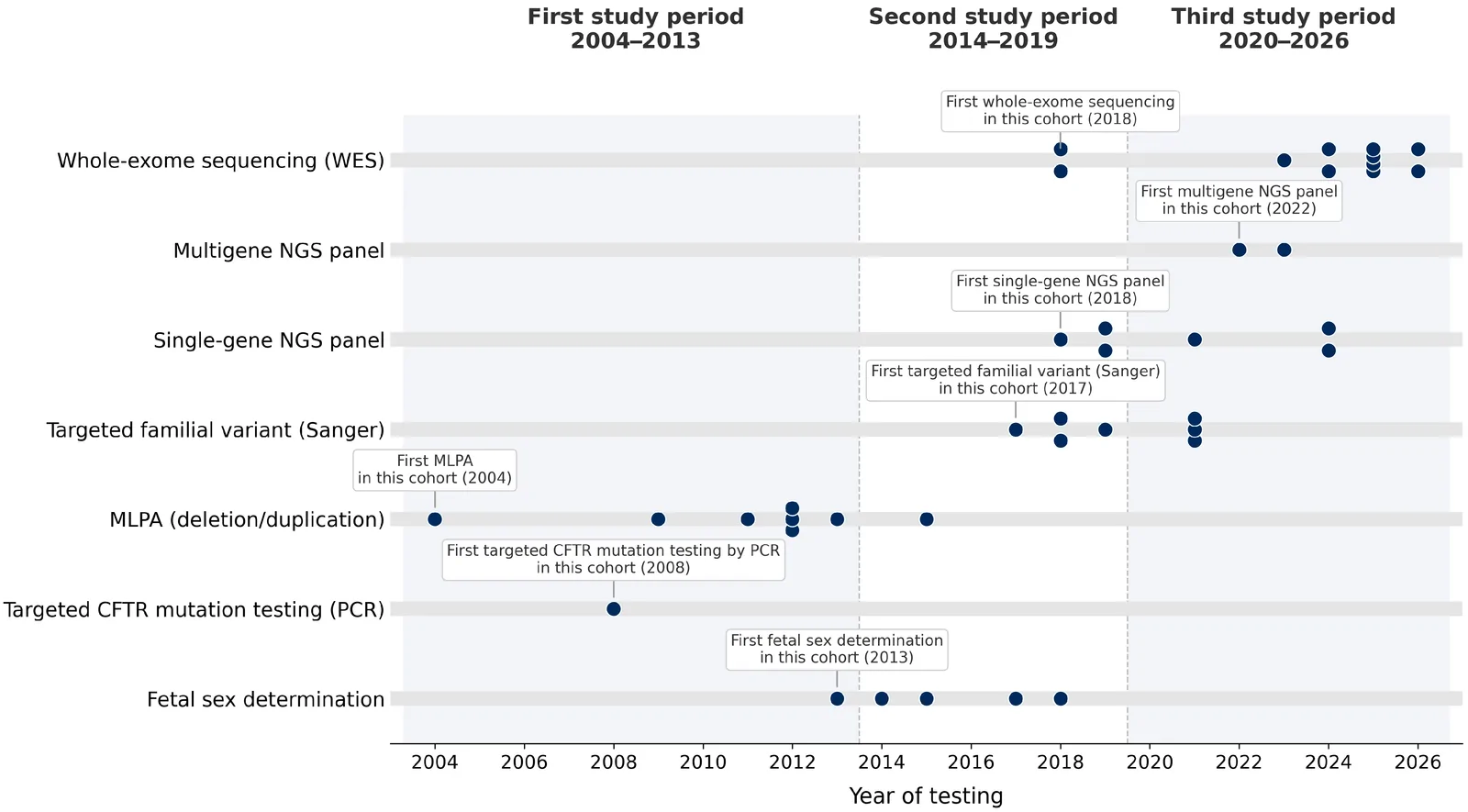
Testing strategies used within the diagnostic pathway (*n* = 40), by year of testing. Each point represents one pregnancy; vertical offset separates points tested in the same year using the same strategy. Vertical dashed lines delimit the three study periods (2004–2013, 2014–2019, and 2020–2026). Annotations indicate the year each strategy was first represented in this cohort. The cfDNA-based monogenic screening pathway (*n* = 12), available only from the third study period onward, is reported separately (Section 3.1) and is not included in this figure.

The testing strategies represented within the diagnostic pathway varied across the three study periods (Figure 3). MLPA was the earliest molecular strategy represented in this cohort, with the first case recorded in 2004. Targeted *CFTR* mutation testing by PCR was first represented in this cohort in 2008, followed by fetal sex determination in 2013. Targeted familial Sanger sequencing was first represented in this cohort in 2017, while single-gene NGS panels and WES were first represented in this cohort in 2018. Multigene NGS panels were first represented in this cohort in 2022.

In the first study period (2004–2013), three testing strategies were represented: MLPA, targeted *CFTR* mutation testing by PCR, and fetal sex determination. From the second study period (2014–2019) onward, the range of testing strategies represented in this cohort included targeted familial Sanger sequencing, single-gene NGS panels, and WES, alongside previously used approaches.

In the later study periods, broader genomic approaches coexisted with continued use of targeted testing for pregnancies with a known familial pathogenic variant.

### 3.4. Molecular Findings, Incidental Findings and Diagnostic Outcomes

The molecular investigations performed during the study period identified a broad spectrum of disease-associated genes and molecular findings, ranging from targeted confirmation or exclusion of known familial variants to broader genomic findings obtained through prenatal whole-exome sequencing. The molecular spectrum, diagnostic categories, incidental findings, and overall diagnostic outcomes are summarized below.

#### 3.4.1. Disease and Gene Spectrum

Of the 40 pregnancies in the diagnostic pathway, 34 underwent fetal molecular evaluation, while in six pregnancies no further molecular testing was performed following fetal sex determination. The 34 molecularly evaluated pregnancies contributed to the gene-spectrum analysis. Three additional records excluded from the analytic pregnancy cohort, including one preconceptional investigation and two postnatal index cases, were retained in Table S2 solely as reference familial records to document molecular context and were excluded from the gene-spectrum analysis.

Four families contributed more than one pregnancy to the diagnostic pathway, reflecting repeated prenatal evaluation across successive pregnancies within the same family. Two of these families were investigated repeatedly for familial *DMD*-related X-linked risk, one family underwent repeated testing for *CFTR* and *HFE*, and one family underwent repeated multigene evaluation involving *TRNT1*, *SERPINC1*, and *C7*. These recurrent familial investigations are identified by matching superscript letters in Table S2 and were retained as separate pregnancies in the analysis because each represented an independent prenatal diagnostic episode.

The resulting gene-spectrum analysis comprised 38 primary gene-level findings involving 19 distinct genes from the 34 prenatally investigated pregnancies. *CFTR* (*n* = 10) and *DMD* (*n* = 6) were the most frequently represented genes, followed by *C7*, *COL7A1*, *GJB2*, *TRNT1*, and *TUBA1A* (*n* = 2 each). The remaining primary findings involved *CENPJ*, *COL1A1*, *DHCR7*, *HFE*, *HPRT1*, *LIFR*, *NF1*, *PKHD1*, *SMN1*, *SMPD1*, *SERPINC1*, and *TAB2* (*n* = 1 each).

Two pregnancies from the same family were investigated for both *CFTR* and *HFE*. Multiple primary gene-level findings were present in three pregnancies: one involving *TRNT1*, *SERPINC1*, and *C7*; one involving *C7* and *TRNT1*; and one involving *TUBA1A* and *GJB2*. Ten additional findings involving *CAPN3*, *CFTR*, *DNAH7*, *GJB2*, *HFE*, *MYH7*, *RNPC3*, *SERPINC1*, and *TBX1* are described separately in Section 3.4.4 and were not counted as independent primary findings in the gene-spectrum analysis (Table S2).

#### 3.4.2. Molecular Findings

Among the 40 pregnancies in the diagnostic pathway, 34 underwent fetal molecular evaluation. Of these, 44.1% were unaffected, 26.5% had carrier-only outcomes, 20.6% had a confirmed disease-causing finding, and 8.8% had non-classic findings without a confirmed molecular diagnosis. In the remaining six pregnancies, no further molecular testing was performed following fetal sex determination for X-linked risk.

Ultrasound abnormalities were the indication for genetic testing in nine pregnancies. Six underwent *CFTR*-related testing: one had a disease-confirming finding, while five had no disease-confirming *CFTR* genotype. Of the three non-*CFTR* cases, one with suspected polycystic kidney disease had a disease-confirming compound heterozygous *PKHD1* genotype. The other two were investigated by WES and yielded molecular findings of uncertain clinical significance that did not establish a definitive molecular diagnosis. One fetus with growth restriction and micrognathia had compound heterozygous *CENPJ* variants as the primary finding, with additional *RNPC3* and *MYH7* findings; the other, with Ebstein anomaly, had *TAB2* as the main candidate gene, with additional *DNAH7* and *GJB2* findings (Table S2).

Within the cfDNA-based monogenic screening pathway (*n* = 12), 100% had a negative result using the respective assay available at the time of testing (Section 2.3). The diagnostic and cfDNA-based pathways were analyzed separately and were not combined into a single diagnostic yield.

#### 3.4.3. Prenatal Whole-Exome Sequencing Findings

Whole-exome sequencing was performed in 11 pregnancies, all investigated during the second and third study periods. Outcomes comprised four confirmed disease-causing findings (36.4%), two carrier-only outcomes (18.2%), three pregnancies classified as unaffected (27.3%), and two with non-classic molecular findings (18.2%). Additional molecular findings were also identified in several WES-investigated pregnancies and are described separately in Section 3.4.4.

#### 3.4.4. Incidental and Additional Findings

Incidental or additional molecular findings beyond the primary testing indication were identified in six pregnancies with fetal molecular evaluation, comprising 10 additional gene-level findings. In two pregnancies from the same family investigated for *CFTR* and *HFE*, the additional findings comprised exclusion of the familial *CFTR* variant in one pregnancy and heterozygous *HFE* carrier status in the other. Additional findings in the remaining pregnancies involved *TBX1* and *CAPN3*; *RNPC3* and *MYH7*; *SERPINC1* and *CAPN3*; and *DNAH7* and *GJB2*, respectively. These findings were interpreted according to their molecular classification and relationship to the primary testing indication (Table S2).

#### 3.4.5. Diagnostic Outcomes

**Figure 4:**
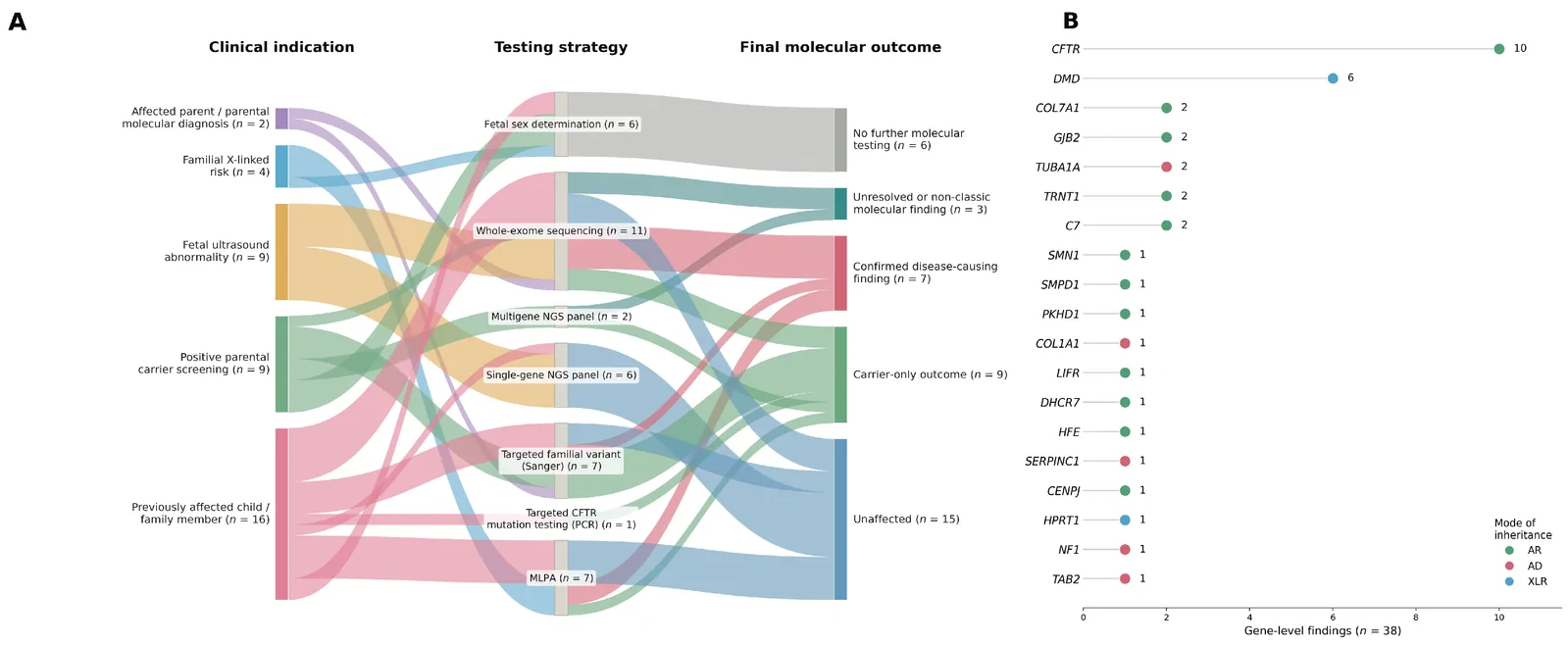
Molecular outcomes and gene spectrum within the diagnostic pathway. (**A**) Clinical indications, testing strategies, and final molecular outcomes among the 40 pregnancies in the diagnostic pathway. Flow colors indicate the corresponding clinical indication and final molecular outcome categories. (**B**)Gene spectrum comprising 38 primary gene-level findings across 19 distinct genes from 34 prenatally investigated pregnancies. The three reference familial findings and the 10 additional findings listed in Table S2 were excluded from the gene-spectrum analysis. Colors indicate mode of inheritance (AR, autosomal recessive; AD, autosomal dominant; XLR, X-linked recessive).

Final molecular outcomes within the diagnostic pathway (*n* = 40) are summarized in Figure 4A: 15 pregnancies (37.5%) were unaffected, nine (22.5%) had carrier-only outcomes, seven (17.5%) had a confirmed disease-causing finding, three (7.5%) had non-classic molecular findings, and six (15.0%) underwent no further molecular testing following fetal sex determination. Among the 34 pregnancies with fetal molecular evaluation, the corresponding spectrum of 38 primary gene-level findings across 19 distinct genes is shown in Figure 4B. Within the separate cfDNA-based monogenic screening pathway (*n* = 12), all pregnancies had a negative screening result.

### 3.5. Clinical Interpretation and Genetic Counseling

Molecular findings were interpreted according to family history, inheritance pattern, fetal phenotype, and recurrence risk.

#### 3.5.1. Interpretation of Molecular Findings by Category

Among the 34 pregnancies with fetal molecular evaluation, seven had a confirmed disease-causing finding, nine had carrier-only outcomes, fifteen were classified as unaffected, and three had non-classic molecular findings. In a further six pregnancies at risk for X-linked disorders, identification of a female fetus resulted in no further molecular testing. Within the separate cfDNA-based monogenic screening pathway (*n* = 12), all pregnancies had a negative screening result, interpreted within the limitations of a screening test.

#### 3.5.2. Variants of Uncertain Significance

In seven pregnancies, *CFTR* TG/T poly-T intronic variants were identified and interpreted according to the overall fetal genotype, family history, and segregation data. Six were not considered diagnostic of cystic fibrosis. One homozygous low-penetrance finding was classified as a non-classic molecular outcome rather than as a disease-confirming genotype. This finding has been primarily associated with *CFTR*-related male infertility; however, its potential clinical relevance was not applicable in this pregnancy because the fetus was female. Additional VUSs identified through broader genomic testing were interpreted in the context of the primary molecular and phenotypic findings.

#### 3.5.3. Genomic Testing and Additional Findings

Incidental or additional molecular findings were identified in six pregnancies within the diagnostic pathway. These included secondary, incidental, and parental findings requiring case-specific interpretation. Detailed findings are provided in Table S2.

#### 3.5.4. Implications for Prenatal Genetic Counseling

Genetic counseling was individualized according to the molecular finding, inheritance pattern, fetal phenotype, and recurrence risk. Counseling addressed prognosis and reproductive options for affected pregnancies with confirmed disease-causing findings, future reproductive implications of carrier-only outcomes, residual risk after negative cfDNA screening, and case-specific interpretation of non-classic molecular findings and additional findings. The counseling implications by molecular outcome are summarized in Table 2.

## 4. Discussion

### 4.1. Principal Findings

This 22-year, single-center study describes how prenatal molecular diagnosis for suspected monogenic disorders changed within a private outpatient medical genetics practice in Western Romania, from predominantly targeted approaches to a broader range of strategies including gene panels, whole-exome sequencing (WES), and cfDNA-based monogenic screening. Three main changes were observed: referral indications broadened from predominantly family-history-based testing to include carrier screening and fetal ultrasound abnormalities; diagnostic strategies expanded, coexisting with rather than replacing targeted familial testing; and interpretive and counseling complexity increased, particularly for incidental, additional, and uncertain findings. As a retrospective descriptive series, this study documents changes observed within a single practice and was not designed to formally compare diagnostic strategies, assess their diagnostic performance, or establish that any strategy improved clinical outcomes.

### 4.2. Evolution of Prenatal Molecular Diagnosis

Targeted testing for a known familial variant remained an important strategy when a familial pathogenic variant was already established (Section 3.3), offering a focused and readily interpretable approach. This underscores the importance of documenting family history and previous molecular diagnoses during prenatal genetic counseling.

The increasing contribution of carrier screening as a referral indication in this cohort (Section 3.2) is consistent with evidence that expanded carrier screening identifies couples at reproductive risk for autosomal recessive and X-linked conditions, including those without a prior family history [3,4].

Fetal ultrasound abnormalities also became a more frequent referral indication and coincided with the introduction of prenatal WES in this cohort. Large prospective studies have demonstrated additional diagnostic findings from prenatal exome sequencing in fetuses with structural anomalies after non-diagnostic chromosomal testing [6,7], while rapid prenatal exome sequencing has also been reported to influence pregnancy management [16]. The gene spectrum observed in our cohort, dominated by *CFTR* and *DMD*, reflects conditions frequently encountered in reproductive and prenatal genetic testing; prenatal approaches targeting *DMD* [17] and spinal muscular atrophy (*SMN1*) [18] further illustrate the application of molecular testing to X-linked and severe recessive disorders.

Incidental or additional molecular findings were identified in 6 of 40 pregnancies within the diagnostic pathway (15.0%). Similar interpretive challenges related to incidental and secondary findings have been reported following exome sequencing [19]. Disclosure preferences may vary among patients and families [20], supporting individualized interpretation within established reporting frameworks and ACMG guidance for secondary findings [21,22]. VUSs similarly required case-specific interpretation, particularly *CFTR* TG/T poly-T variants in this cohort.

cfDNA-based monogenic screening was introduced in this practice in 2024 as a distinct, non-diagnostic screening pathway [5,23]. Its use alongside continued invasive testing illustrates the coexistence of screening and diagnostic approaches rather than their interchangeability. This distinction is consistent with reports extending cfDNA-based approaches beyond aneuploidy to selected monogenic disorders [5,24].

Referral patterns and available testing strategies changed concurrently over the study period and cannot be readily disentangled. The sequential introduction of gene panels, WES, and cfDNA-based screening also reflects their increasing availability over time, while growth in referrals may have been influenced by clinic growth, referral networks, affordability, and awareness of genetic testing. Without a comparator group or population denominator, this descriptive series cannot determine whether newer strategies improved diagnostic performance or pregnancy outcomes; it documents the testing strategies used, their clinical indications, and the molecular findings observed within this practice.

### 4.3. A Single-Center Experience in Western Romania

This series was conducted at a single private outpatient medical genetics practice in Western Romania, a region without a dedicated public prenatal genomics center for most of the study period. Throughout the study period, prenatal molecular testing depended on a combination of Romanian public and private laboratories and international reference laboratories, rather than on a single integrated national pathway. Longitudinal cohorts documenting this trajectory from within routine clinical practice remain uncommon in Romania and the wider region.

The value of this series lies mainly in its longitudinal continuity: it documents how successive diagnostic technologies were integrated within the same clinical practice over 22 years, rather than comparing unrelated cohorts from different institutions. Despite the modest cohort size, a consequence of the narrow inclusion criteria described in Section 2.1 (see also Section 4.5), the study captures a highly selected diagnostic pathway rarely described longitudinally in Romania and Central and Eastern Europe.

Advanced prenatal molecular testing in this setting has been provided through both national and international laboratories, reflecting a pragmatic and decentralized model of access; however, this single-center experience should not be considered representative of prenatal genetic services throughout Romania. Published longitudinal data on prenatal monogenic diagnosis from Romania remain limited, in the broader European context of disparities in access to clinical genetics services [8,25].

Recent developments in Romanian population-level genetic screening add further context to these findings. In February 2026, the Romanian Ministry of Health launched a National Neonatal Screening Registry, with plans to expand newborn screening from 3 to 22 genetic and metabolic conditions [26]. This program addresses postnatal, population-wide metabolic and genetic screening and is not equivalent to prenatal diagnosis. Nevertheless, it reflects increasing institutional attention to genetic and genomic medicine in Romania, providing broader national context for the prenatal molecular testing described in the present series.

### 4.4. Clinical Implications

These findings carry practical implications for prenatal genetic practice. Test selection should be guided primarily by clinical context, including family history, fetal phenotype, and carrier status, rather than by the most comprehensive technology available. The continued relevance of targeted testing supports systematic family-history-taking and cascade testing when a familial pathogenic variant is known, before broader panel- or exome-based testing is considered. Second-trimester fetal ultrasound may identify structural anomalies that warrant broader genomic investigation, including WES when prior genetic testing is non-diagnostic [6,7]. Pre-test counseling should address the possibility of VUS, incidental findings, and additional findings. Finally, cfDNA-based monogenic screening requires a clear distinction between screening and diagnostic results in counseling, including discussion of residual risk and the need for confirmatory testing following a positive screening result [5,23].

### 4.5. Strengths, Limitations and Future Perspectives

This study has several strengths: it is one of the longer single-center prenatal molecular diagnosis series reported from Romania, and one of the few to capture the full clinical pathway, from referral indication through diagnostic strategy to molecular outcome and counseling, within a single, consistently documented cohort. This allows the coexistence of targeted and genomic approaches to be observed directly rather than inferred from separate studies.

This study also has several limitations. As a retrospective descriptive series from a single private outpatient practice, the 52 pregnancies represent a highly selected subset of prenatal testing activity, restricted to suspected monogenic indications (Section 2.1). The cohort does not represent a defined catchment population or a complete denominator of pregnancies or referrals seen at the practice and therefore cannot be used to estimate population-level prevalence, incidence, or uptake of prenatal molecular testing. The modest sample size limits comparisons across study periods or subgroups, and the single-center setting limits generalizability to other practices. Referral patterns, technology availability, financial accessibility, and laboratory platforms changed over the 22-year study period and cannot be fully disentangled, limiting inference about the drivers of the observed changes. For the cfDNA-based monogenic screening pathway, confirmatory invasive testing was not systematically available for screen-negative results; therefore, sensitivity, specificity, and overall clinical performance cannot be assessed in this cohort. Technical factors such as maternal cell contamination, a recognized potential confounder in prenatal genetic testing [27], were not systematically assessed and cannot be excluded. Long-term reproductive and developmental outcomes beyond the immediate neonatal period were not systematically available for all pregnancies. Multicenter Romanian studies with standardized data collection and outcome reporting would help establish whether the patterns described here generalize to other regional genetic practices. Prospective evaluation of cfDNA-based monogenic screening, including systematic confirmatory testing where appropriate, together with integration of prenatal molecular testing into national genetic-service networks and registries, represents a further direction for this field.

## 5. Conclusions

Over a 22-year period, prenatal molecular diagnosis for suspected monogenic disorders in this Western Romanian medical genetics practice broadened from predominantly family-history-based testing to include indications arising from carrier screening and fetal phenotype assessment, while testing strategies expanded to include gene panels, whole-exome sequencing, and cfDNA-based monogenic screening as a separate, non-diagnostic pathway. This change was not a simple replacement of older methods: targeted testing remained relevant when a familial pathogenic variant was known, while genomic and screening technologies were used alongside established approaches. Molecular testing confirmed the suspected diagnosis in 17.5% of pregnancies within the diagnostic pathway, while in a further 5%, molecular findings potentially associated with the observed fetal ultrasound abnormalities were identified. The introduction of WES into clinical practice and the broader spectrum of genetic tests offered have increased the potential for identifying incidental or additional findings, which occurred in 17.6% of molecularly evaluated pregnancies and required careful case-specific interpretation. Monogenic cfDNA screening yielded exclusively negative results in this cohort and should be regarded as a complementary screening approach rather than an alternative to diagnostic testing.

Overall, this series illustrates the evolution of prenatal molecular diagnosis over more than two decades of routine clinical practice, from targeted testing guided by family history and specific clinical suspicion to increasingly comprehensive genomic approaches. The expanding range of available technologies has broadened diagnostic possibilities while introducing new challenges in the interpretation and communication of complex molecular findings. These observations support the continued integration of genomic testing into prenatal care within a multidisciplinary framework combining careful phenotypic assessment, appropriate test selection, molecular interpretation, and individualized genetic counseling.

## Figures and Tables

**Table 1:** Baseline characteristics of the overall study cohort and the diagnostic pathway.

Variable	Overall (*n* = 52)	Diagnostic Pathway (*n* = 40)	cfDNA Pathway (*n* = 12)
Maternal age, years, mean ± SD (median; range)	31.7 ± 5.9 (31; 19–46)	29.8 ± 4.8 (30; 19–41)	38.0 ± 4.8 (38; 31–46)
Gestational age at sampling, weeks, median (range)	16 (10–24)	16 (10–24)	11 (10–13)
**Sample type, *n* (%)**			
Amniocentesis	33 (63.5)	33 (82.5)	—
Chorionic villus sampling (CVS)	5 (9.6)	5 (12.5)	—
Maternal blood (cfDNA)	12 (23.1)	—	12 (100)
Non-invasive (fetal sex determination only)	2 (3.8)	2 (5.0)	—
**Study period, *n* (%)**			
2004–2013	9 (17.3)	9 (22.5)	0 (0.0)
2014–2019	14 (26.9)	14 (35.0)	0 (0.0)
2020–2026	29 (55.8)	17 (42.5)	12 (100)
Previously affected child *, *n* (%)	17 (32.7)	16 (40.0)	1 (8.3)

* Previously affected child is presented here as a baseline cohort characteristic; where it also constituted the primary clinical indication for referral, it is discussed again in Section 3.2.

**Table 2:** Clinical interpretation and genetic counseling implications by molecular outcome and clinical pathway.

Molecular Outcome	Molecular Interpretation	Recurrence-Risk Assessment	Counseling Implications	Additional Evaluation
**Diagnostic pathway ** ** (*n* = 40)**				
Unaffected (*n* = 15)	No disease-confirming molecular finding	Case-specific according to the familial or clinical context	Residual risk explained	Follow-up according to clinical indication
Carrier-only outcome (*n* = 9)	Carrier status only; disease not expected	Disease excluded for the current pregnancy for the condition under investigation	Future reproductive implications discussed	Partner/family testing if appropriate
Confirmed disease-causing finding (*n* = 7)	Disease confirmed	Inheritance-specific	Prognosis and reproductive counseling	Family segregation if indicated
No further molecular testing following fetal sex determination (*n* = 6)	Female fetus; no further molecular testing	Risk for the X-linked condition considered excluded for the current pregnancy	Implications of fetal sex explained	None required
Non-classic molecular finding (*n* = 3)	Molecular finding not diagnostic or not formally confirmed	Case-specific; dependent on available molecular and segregation evidence	Counseling under uncertainty	Reevaluation, segregation studies, or additional testing if appropriate
**cfDNA-based monogenic screening pathway (*n* = 12)**				
Negative cfDNA screening result (*n* = 12)	Negative screening result; screened conditions not detected	Residual risk remains	Screening limitations and confirmatory testing discussed	Diagnostic testing if clinically indicated
**Cross-cutting finding**				
Incidental or additional molecular finding (*n* = 6)	Context-dependent (additional, incidental, or parental)	Case-specific	Individualized counseling	Family evaluation or reevaluation if clinically relevant

## Data Availability

The original contributions presented in this study are included in the article/Supplementary Materials. Further inquiries can be directed to the corresponding authors.

## Supplementary Materials

The following supporting information can be downloaded at: https://www.mdpi.com/article/10.3390/medsci14050548/s1, Table S1: Individual characteristics of pregnancies included in the analytic cohort; Table S2. Gene-level and molecular findings identified within the diagnostic pathway and reference familial records.

## Abbreviations

The following abbreviations are used in this manuscript:

ACMG	American College of Medical Genetics and Genomics
AD	Autosomal Dominant
AMP	Association for Molecular Pathology
AR	Autosomal Recessive
BGI	Beijing Genomics Institute
cfDNA	Cell-Free DNA
CMA	Chromosomal Microarray Analysis
CVS	Chorionic Villus Sampling
DNA	Deoxyribonucleic Acid
FISH	Fluorescence In Situ Hybridization
HGVS	Human Genome Variation Society
MLPA	Multiplex Ligation-Dependent Probe Amplification
NGS	Next-Generation Sequencing
NIFTY	Non-Invasive Fetal TrisomY
NIPT	Non-Invasive Prenatal Testing
PCR	Polymerase Chain Reaction
SD	Standard Deviation
VUS	Variant of Uncertain Significance
WES	Whole-Exome Sequencing
XR	X-linked Recessive
