# Supplementary material for: Twenty-Two Years of Prenatal Testing for Suspected Monogenic Disorders: A Retrospective Single-Center Experience in Western Romania
Source: Med Sci (Basel). 2026 Sep 7;14(5):548. doi: 10.3390/medsci14050548 (PMC13609703; doi:10.3390/medsci14050548)
Supplement: Supplementary file 1 [file medsci-14-00548-s001.zip › Supplementary_Table_S1.pdf]

**Supplementary Table S1.** Individual characteristics of pregnancies included in the analytic cohort

| Study ID | Year | Study period | Maternal age (years) | Gestational age at sampling (weeks) | Clinical pathway | Primary clinical indication | Sample type | Diagnostic or screening strategy | Gene(s) investigated | Molecular finding category | Final diagnostic outcome | Pregnancy/ neonatal outcome | Incidental/ additional finding |
|---|---|---|---|---|---|---|---|---|---|---|---|---|---|
| DP01[a] | 2004 | First study period (2004–2013) | 22 | 14 | Diagnostic pathway | Familial X-linked risk | Chorionic villus sample | MLPA | *DMD* | Familial variant absent | Unaffected fetus | Pregnancy continued; Live birth, unaffected | None reported |
| DP02 | 2008 | | 30 | 16 | Diagnostic pathway | Previously affected child or family member | Amniotic fluid | Targeted CFTR mutation testing by PCR | *CFTR* | Carrier status | Carrier-only outcome | Pregnancy continued; Live birth, healthy carrier | None reported |
| DP03 | 2009 | | 29 | 22 | Diagnostic pathway | Previously affected child or family member | Chorionic villus sample | MLPA | *DMD* | Familial variant absent | Unaffected fetus | Pregnancy continued; Live birth, unaffected | None reported |
| DP04[a] | 2011 | | 28 | 16 | Diagnostic pathway | Positive parental carrier screening | Chorionic villus sample; amniotic fluid | Fetal sex determination | *DMD* | No further molecular testing | No further molecular testing following fetal sex determination | Pregnancy continued (46,XX karyotype confirmed on repeat amniocentesis; DMD not tested) | Chromosomal (trisomy on CVS karyotype, not confirmed on repeat amniocentesis) |
| DP05[b] | 2012 | | 30 | 16 | Diagnostic pathway | Previously affected child or family member | Amniotic fluid | MLPA | *DMD* | Pathogenic /likely pathogenic finding | Confirmed disease-causing finding | Pregnancy medically terminated | None reported |
| DP06 | 2012 | | 25 | 16 | Diagnostic pathway | Familial X-linked risk | Amniotic fluid | MLPA | *DMD* | Familial variant absent | Unaffected fetus | Pregnancy continued; Live birth, unaffected | None reported |

| Study ID | Year | Study period | Maternal age (years) | Gestational age at sampling (weeks) | Clinical pathway | Primary clinical indication | Sample type | Diagnostic or screening strategy | Gene(s) investigated | Molecular finding category | Final diagnostic outcome | Pregnancy/ neonatal outcome | Incidental/ additional finding |
|---|---|---|---|---|---|---|---|---|---|---|---|---|---|
| DP07 | 2012 | | 24 | 17 | Diagnostic pathway | Familial X-linked risk | Amniotic fluid | MLPA | *DMD* | Familial variant absent | Unaffected fetus | Pregnancy continued; Live birth, unaffected | None reported |
| DP08[b] | 2013 | | 30 | 13 | Diagnostic pathway | Previously affected child or family member | Chorionic villus sample | Fetal sex determination | N/A - sex determination only; Fetal sex: FEMALE (46,XX); DMD risk excluded (X-linked recessive) | No further molecular testing | No further molecular testing following fetal sex determination | Pregnancy continued; Live birth, unaffected | None reported |
| DP09 | 2013 | | 29 | 16 | Diagnostic pathway | Previously affected child or family member | Chorionic villus sample | MLPA | *DMD* | Pathogenic /likely pathogenic finding | Confirmed disease-causing finding | Pregnancy medically terminated | None reported |
| DP10 | 2014 | **Second study period (2014–2019)** | 33 | 10 | Diagnostic pathway | Positive parental carrier screening | Non-invasive fetal sex determination | Fetal sex determination | N/A - sex determination only; Fetal sex: FEMALE (46,XX); Haemophilia A risk excluded (X-linked recessive) | No further molecular testing | No further molecular testing following fetal sex determination | Pregnancy continued; Live birth, unaffected | None reported |
| DP12 | 2015 | | 30 | 16 | Diagnostic pathway | Previously affected child or family member | Amniotic fluid | MLPA | *SMN1* | Carrier status | Carrier-only outcome | Pregnancy continued; Live birth, healthy carrier | None reported |

| Study ID | Year | Study period | Maternal age (years) | Gestational age at sampling (weeks) | Clinical pathway | Primary clinical indication | Sample type | Diagnostic or screening strategy | Gene(s) investigated | Molecular finding category | Final diagnostic outcome | Pregnancy/ neonatal outcome | Incidental/ additional finding |
|---|---|---|---|---|---|---|---|---|---|---|---|---|---|
| DP11 | 2015 | | 32 | 10 | Diagnostic pathway | Familial X-linked risk | Non-invasive fetal sex determination | Fetal sex determination | N/A - sex determination only; Fetal sex: FEMALE (46,XX); DMD risk excluded (X-linked recessive) | No further molecular testing | No further molecular testing following fetal sex determination | Pregnancy continued; Live birth, unaffected | None reported |
| DP13 | 2017 | | 31 | 16 | Diagnostic pathway | Previously affected child or family member | Amniotic fluid | Targeted familial variant testing by Sanger sequencing | *COL7A1* | Carrier status | Carrier-only outcome | Pregnancy continued; Live birth, healthy carrier | None reported |
| DP14[b] | 2017 | | 34 | 16 | Diagnostic pathway | Previously affected child or family member | Amniotic fluid | Fetal sex determination | N/A - sex determination only; Fetal sex: FEMALE (46,XX); DMD risk excluded (X-linked recessive) | No further molecular testing | No further molecular testing following fetal sex determination | Pregnancy continued; Live birth, unaffected | None reported |
| DP17 | 2018 | | 31 | 24 | Diagnostic pathway | Fetal ultrasound abnormality | Amniotic fluid | Whole-exome sequencing | *PKHD1* | Pathogenic /likely pathogenic finding | Confirmed disease-causing finding | Pregnancy continued; neonatal death on day 2. Autopsy showed polycystic kidneys and hepatic lesions. | None reported |

| Study ID | Year | Study period | Maternal age (years) | Gestational age at sampling (weeks) | Clinical pathway | Primary clinical indication | Sample type | Diagnostic or screening strategy | Gene(s) investigated | Molecular finding category | Final diagnostic outcome | Pregnancy/ neonatal outcome | Incidental/ additional finding |
|---|---|---|---|---|---|---|---|---|---|---|---|---|---|
| DP15 | 2018 | | 24 | 18 | Diagnostic pathway | Positive parental carrier screening | Amniotic fluid | Targeted familial variant testing by Sanger sequencing | *SMPD1* | Pathogenic /likely pathogenic finding | Confirmed disease-causing finding | Pregnancy continued; Live birth, affected | None reported |
| DP16 | 2018 | | 31 | 20 | Diagnostic pathway | Fetal ultrasound abnormality | Amniotic fluid | Whole-exome sequencing | *CFTR* | Pathogenic /likely pathogenic finding | Confirmed disease-causing finding | Pregnancy continued; Live birth, affected | None reported |
| DP19 | 2018 | | 30 | 16 | Diagnostic pathway | Affected parent with a known autosomal dominant condition | Amniotic fluid | Targeted familial variant testing by Sanger sequencing | *COL1A1* | Familial variant absent | Unaffected fetus | Pregnancy continued; Live birth, unaffected | None reported |
| DP20 | 2018 | | 28 | 16 | Diagnostic pathway | Fetal ultrasound abnormality | Amniotic fluid | Single-gene NGS panel | *CFTR* | Familial variant absent | Unaffected fetus | Pregnancy continued; Live birth, unaffected | None reported |
| DP18[b] | 2018 | | 30 | 16 | Diagnostic pathway | Positive parental carrier screening | Amniotic fluid | Fetal sex determination | N/A - sex determination only; Fetal sex: FEMALE (46,XX); DMD risk excluded (X-linked recessive) | No further molecular testing | No further molecular testing following fetal sex determination | Pregnancy continued; Live birth, unaffected | None reported |
| DP21 | 2019 | | 20 | 16 | Diagnostic pathway | Fetal ultrasound abnormality | Amniotic fluid | Single-gene NGS panel | *CFTR* | Familial variant absent | Unaffected fetus | Pregnancy continued; Live birth, unaffected | None reported |

| Study ID | Year | Study period | Maternal age (years) | Gestational age at sampling (weeks) | Clinical pathway | Primary clinical indication | Sample type | Diagnostic or screening strategy | Gene(s) investigated | Molecular finding category | Final diagnostic outcome | Pregnancy/ neonatal outcome | Incidental/ additional finding |
|---|---|---|---|---|---|---|---|---|---|---|---|---|---|
| DP22 | 2019 | | 19 | 20 | Diagnostic pathway | Fetal ultrasound abnormality | Amniotic fluid | Single-gene NGS panel | *CFTR* | No disease-confirming molecular finding | Unaffected | Pregnancy continued; Live birth, unaffected | None reported |
| DP23 | 2019 | | 41 | 16 | Diagnostic pathway | Previously affected child or family member | Amniotic fluid | Targeted familial variant testing by Sanger sequencing | *COL7A1* | Familial variant absent | Unaffected fetus | Pregnancy continued; Live birth, unaffected | None reported |
| DP24 | 2021 | **Third study period (2020–2026)** | **31** | 16 | Diagnostic pathway | Positive parental carrier screening | Amniotic fluid | Targeted familial variant testing by Sanger sequencing | *GJB2* | Carrier status | Carrier-only outcome | Pregnancy continued; Live birth, healthy carrier | None reported |
| DP25 | 2021 | | 28 | 16 | Diagnostic pathway | Previously affected child or family member | Amniotic fluid | Targeted familial variant testing by Sanger sequencing | *LIFR* | Carrier status | Carrier-only outcome | Pregnancy continued; Live birth, healthy carrier | None reported |
| DP26 | 2021 | | 40 | 16 | Diagnostic pathway | Positive parental carrier screening | Amniotic fluid | Targeted familial variant testing by Sanger sequencing | *DHCR7* | Carrier status | Carrier-only outcome | Pregnancy continued; Live birth, healthy carrier | None reported |
| DP27 | 2021 | | 19 | 23 | Diagnostic pathway | Fetal ultrasound abnormality | Amniotic fluid | Single-gene NGS panel | *CFTR* | Familial variant absent | Unaffected fetus | Pregnancy continued; Live birth, unaffected | None reported |

| Study ID | Year | Study period | Maternal age (years) | Gestational age at sampling (weeks) | Clinical pathway | Primary clinical indication | Sample type | Diagnostic or screening strategy | Gene(s) investigated | Molecular finding category | Final diagnostic outcome | Pregnancy/ neonatal outcome | Incidental/ additional finding |
|---|---|---|---|---|---|---|---|---|---|---|---|---|---|
| DP28[c] | 2022 | | 30 | 17 | Diagnostic pathway | Positive parental carrier screening | Amniotic fluid | Multi-gene NGS panel | *CFTR; HFE* | Carrier status | Carrier-only outcome | Pregnancy continued; Live birth, healthy carrier | CFTR familial variant excluded |
| DP29 | 2023 | | 33 | 16 | Diagnostic pathway | Previously affected child or family member | Amniotic fluid | Whole-exome sequencing | *TUBA1A* | Familial variant absent | Unaffected fetus | Pregnancy continued; Live birth, unaffected | None reported |
| DP30[c] | 2023 | | 32 | 16 | Diagnostic pathway | Positive parental carrier screening | Amniotic fluid | Multi-gene NGS panel | *CFTR; HFE* | Homozygous low-penetrance finding | Unresolved or non-classic molecular finding | Pregnancy continued; Live birth, healthy carrier | HFE carrier finding |
| cfDNA 01 | 2024 | | 33 | 11 | cfDNA-based monogenic screening pathway | Routine screening, apparently normal pregnancy | Maternal blood | NIFTY-MONO (18-gene panel; 2,038 P/LP variants) | No clinically relevant gene-level finding | Negative cfDNA screening | Negative cfDNA screening | Pregnancy continued | None reported |
| cfDNA 02 | 2024 | | 39 | 11 | cfDNA-based monogenic screening pathway | Previously affected child (CHD7-related CHARGE syndrome) | Maternal blood | NIFTY-MONO (18-gene panel; 2,038 P/LP variants) | No clinically relevant gene-level finding | Negative cfDNA screening | Negative cfDNA screening | Pregnancy continued; Live birth, unaffected | None reported |
| DP32[d] | 2024 | | 31 | 20 | Diagnostic pathway | Previously affected child or family member | Amniotic fluid | Whole-exome sequencing | *TRNT1; SERPINC1; C7; TBX1; CAPN3* | Pathogenic /likely pathogenic finding | Confirmed disease-causing finding | Pregnancy medically terminated | TBX1 VUS; CAPN3 incidental finding |

| Study ID | Year | Study period | Maternal age (years) | Gestational age at sampling (weeks) | Clinical pathway | Primary clinical indication | Sample type | Diagnostic or screening strategy | Gene(s) investigated | Molecular finding category | Final diagnostic outcome | Pregnancy/ neonatal outcome | Incidental/ additional finding |
|---|---|---|---|---|---|---|---|---|---|---|---|---|---|
| DP31 | 2024 | | 26 | 16 | Diagnostic pathway | Fetal ultrasound abnormality | Amniotic fluid | Single-gene NGS panel | *CFTR* | Familial variant absent | Unaffected fetus | Pregnancy continued; Live birth, unaffected | None reported |
| DP33 | 2024 | | 33 | 16 | Diagnostic pathway | Previously affected child or family member | Amniotic fluid | Single-gene NGS panel | *CFTR* | Familial variant absent | Unaffected fetus | Pregnancy continued; Live birth, unaffected | None reported |
| cfDNA 03 | 2024 | | 46 | 11 | cfDNA-based monogenic screening pathway | Advanced maternal and paternal age; previous unsuccessful IVF | Maternal blood | NIFTY-MONO (18-gene panel; 2,038 P/LP variants) | No clinically relevant gene-level finding | Negative cfDNA screening | Negative cfDNA screening | Pregnancy continued | None reported |
| cfDNA 04 | 2024 | | 34 | 10 | cfDNA-based monogenic screening pathway | Routine screening; previous ectopic pregnancy | Maternal blood | NIFTY-MONO (18-gene panel; 2,038 P/LP variants) | No clinically relevant gene-level finding | Negative cfDNA screening | Negative cfDNA screening | Pregnancy continued | None reported |
| cfDNA 05 | 2024 | | 39 | 11 | cfDNA-based monogenic screening pathway | Advanced maternal and paternal age | Maternal blood | NIFTY-MONO (18-gene panel; 2,038 P/LP variants) | No clinically relevant gene-level finding | Negative cfDNA screening | Negative cfDNA screening | Pregnancy continued | None reported |
| DP34 | 2024 | | 29 | 21 | Diagnostic pathway | Fetal ultrasound abnormality | Amniotic fluid | Whole-exome sequencing | *CENPJ*; *RNPC3*; *MYH7* | Unresolved molecular finding | Unresolved or non-classic molecular finding | Pregnancy medically terminated | Additional findings in RNPC3 and MYH7 |

| Study ID | Year | Study period | Maternal age (years) | Gestational age at sampling (weeks) | Clinical pathway | Primary clinical indication | Sample type | Diagnostic or screening strategy | Gene(s) investigated | Molecular finding category | Final diagnostic outcome | Pregnancy/ neonatal outcome | Incidental/ additional finding |
|---|---|---|---|---|---|---|---|---|---|---|---|---|---|
| cfDNA 06 | 2024 | | 31 | 10 | cfDNA-based monogenic screening pathway | Routine screening; previous pregnancy loss; family history of Coffin–Siris syndrome | Maternal blood | NIFTY-MONO (155-gene panel; 6,246 P/LP variants) | No clinically relevant gene-level finding | Negative cfDNA screening | Negative cfDNA screening | Pregnancy continued | None reported |
| cfDNA 07 | 2024 | | 34 | 10 | cfDNA-based monogenic screening pathway | Routine screening; partner with prior chemo/radiotherapy | Maternal blood | NIFTY-MONO (155-gene panel; 6,246 P/LP variants) | No clinically relevant gene-level finding | Negative cfDNA screening | Negative cfDNA screening | Pregnancy continued | None reported |
| DP35 | 2025 | | 34 | 16 | Diagnostic pathway | Previously affected child | Amniotic fluid | Whole-exome sequencing | *HPRT1* | Familial variant absent | Unaffected fetus | Pregnancy continued; Live birth, unaffected | None reported |
| DP36[d] | 2025 | | 32 | 16 | Diagnostic pathway | Previously affected child | Amniotic fluid | Whole-exome sequencing | *C7*; *TRNT1*; *SERPINC1*; *CAPN3* | Carrier status | Carrier-only outcome | Pregnancy continued; Live birth, healthy carrier | Additional findings in SERPINC1 and CAPN3 |
| DP37 | 2025 | | 30 | 16 | Diagnostic pathway | Affected parent with a known autosomal dominant condition | Amniotic fluid | Whole-exome sequencing | *NF1* | Familial variant absent | Unaffected fetus | Pregnancy continued; Live birth, unaffected | None reported |
| DP38 | 2025 | | 33 | 17 | Diagnostic pathway | Positive parental carrier screening | Amniotic fluid | Whole-exome sequencing | *CFTR* | Carrier status | Carrier-only outcome | Pregnancy continued; Live birth, healthy carrier | None reported |

| Study ID | Year | Study period | Maternal age (years) | Gestational age at sampling (weeks) | Clinical pathway | Primary clinical indication | Sample type | Diagnostic or screening strategy | Gene(s) investigated | Molecular finding category | Final diagnostic outcome | Pregnancy/ neonatal outcome | Incidental/ additional finding |
|---|---|---|---|---|---|---|---|---|---|---|---|---|---|
| cfDNA 08 | 2025 | | 39 | 10 | cfDNA-based monogenic screening pathway | Advanced maternal and paternal age | Maternal blood | NIFTY-MONO (155-gene panel; 6,246 P/LP variants) | No clinically relevant gene-level finding | Negative cfDNA screening | Negative cfDNA screening | Pregnancy continued | None reported |
| cfDNA 09 | 2025 | | 36 | 11 | cfDNA-based monogenic screening pathway | Advanced maternal and paternal age | Maternal blood; amniotic fluid (confirmatory) | NIFTY-MONO (155-gene panel; 6,246 P/LP variants) | No clinically relevant gene-level finding | Negative screening result | Negative cfDNA screening | Pregnancy continued | None reported |
| cfDNA 10 | 2025 | | 46 | 10 | cfDNA-based monogenic screening pathway | Fetal ultrasound abnormality in previous pregnancy (ventriculomegaly, cerebellar hypoplasia, talipes); IVF pregnancy | Maternal blood | NIFTY-MONO (expanded 155-gene panel; >22,500 P/LP variants across 218 monogenic disorders) | No clinically relevant gene-level finding | Negative cfDNA screening | Negative cfDNA screening | Pregnancy continued | None reported |
| DP39 | 2026 | | 32 | 21 | Diagnostic pathway | Previously affected child or family member | Amniotic fluid | Whole-exome sequencing | *TUBA1A; GJB2* | Pathogenic/likely pathogenic finding | Confirmed disease-causing finding | Pregnancy medically terminated at 23 weeks + 5 days | None reported |
| DP40 | 2026 | | 37 | 20 | Diagnostic pathway | Fetal ultrasound abnormality | Amniotic fluid | Whole-exome sequencing | *TAB2*; *DNAH7*; *GJB2* | Unresolved molecular finding | Unresolved or non-classic molecular finding | Pregnancy medically terminated | Additional findings in DNAH7 and GJB2 |

| Study ID | Year | Study period | Maternal age (years) | Gestational age at sampling (weeks) | Clinical pathway | Primary clinical indication | Sample type | Diagnostic or screening strategy | Gene(s) investigated | Molecular finding category | Final diagnostic outcome | Pregnancy/ neonatal outcome | Incidental/ additional finding |
|---|---|---|---|---|---|---|---|---|---|---|---|---|---|
| cfDNA 11 | 2026 | | 38 | 13 | cfDNA-based monogenic screening pathway | Fetal ultrasound abnormality in previous pregnancy; suspected tuberous sclerosis | Maternal blood | NIFTY-MONO (expanded 155-gene panel; >22,500 P/LP variants across 218 monogenic disorders) | No clinically relevant gene-level finding | Negative cfDNA screening | Negative cfDNA screening | Pregnancy continued | None reported |
| cfDNA 12 | 2026 | | 41 | 11 | cfDNA-based monogenic screening pathway | Advanced paternal age | Maternal blood | NIFTY-MONO (expanded 155-gene panel; >22,500 P/LP variants across 218 monogenic disorders) | No clinically relevant gene-level finding | Negative cfDNA screening | Negative cfDNA screening | Pregnancy continued | None reported |

**Note:** Study IDs were assigned separately within the diagnostic pathway (DP01–DP40) and the cfDNA-based monogenic screening pathway (cfDNA01–cfDNA12). Pregnancies from the same family are indicated by matching superscript lowercase letters ([a,b,c,d]). Individual pregnancy-level characteristics are presented for all 52 pregnancies included in the analytic cohort.

**Abbreviations:** DP, diagnostic-pathway pregnancy identifier; cfDNA, cell-free DNA; CVS, chorionic villus sampling; MLPA, multiplex ligation-dependent probe amplification; NGS, next-generation sequencing; WES, whole-exome sequencing; PCR, polymerase chain reaction; IVF, in vitro fertilization; N/A, not applicable.
