# Supplementary material for: Twenty-Two Years of Prenatal Testing for Suspected Monogenic Disorders: A Retrospective Single-Center Experience in Western Romania
Source: Med Sci (Basel). 2026 Sep 7;14(5):548. doi: 10.3390/medsci14050548 (PMC13609703; doi:10.3390/medsci14050548)
Supplement: Supplementary file 1 [file medsci-14-00548-s001.zip › Supplementary_Table_S2.pdf]

Supplementary Table S2. Gene-level and molecular findings identified within the diagnostic pathway and reference familial records

Study ID	Year	Clinical indication	Diagnostic strategy	Gene	Mode of inheritance	Variant	Fetal molecular status	Relationship to primary indication	Final clinical interpretation
DP01[a]	2004	Familial X-linked risk	MLPA	*DMD*	XLR	Maternal c.2381-?_5922+?dup (DMD exons 20–41 duplication)	Unaffected non-carrier	Primary finding	Familial variant excluded
DP02	2008	Previously affected child or family member	Targeted CFTR mutation testing by PCR	*CFTR*	AR	c.1521_1523del (p.Phe508del)	Unaffected carrier	Primary finding	Carrier status only; disease not expected
DP03	2009	Previously affected child or family member	MLPA	*DMD*	XLR	Exon 52 deletion	Unaffected non-carrier	Primary finding	Familial variant excluded
RF01	2009	Preconceptional familial molecular diagnosis	Targeted familial variant testing by Sanger sequencing	*NF1*	AD	c.6709C>T (p.Arg2237Ter)	Not applicable	Familial-context reference	Preconceptional familial molecular context
DP04[a]	2011	Positive parental carrier screening	Fetal sex determination	*DMD*	XLR	Maternal c.2381-?_5922+?dup (DMD exons 20–41 duplication)	No further molecular testing	Not included in gene-level analysis	Female fetus; no further molecular testing
DP05[b]	2012	Previously affected child or family member	MLPA	*DMD*	XLR	c.6439-?_7660+?dup (DMD exons 45–52 duplication)	Affected	Primary finding	Disease confirmed
DP06	2012	Familial X-linked risk	MLPA	*DMD*	XLR	Familial DMD rearrangement not identified	Unaffected non-carrier	Primary finding	Familial variant excluded
DP07	2012	Familial X-linked risk	MLPA	*DMD*	XLR	Familial DMD rearrangement not identified	Unaffected non-carrier	Primary finding	Familial variant excluded
DP08[b]	2013	Previously affected child or family member	Fetal sex determination	*DMD*	XLR	Familial DMD exons 45–52 duplication	No further molecular testing	Not included in gene-level analysis	Female fetus; no further molecular testing
DP09	2013	Previously affected child or family member	MLPA	*DMD*	XLR	DMD exons 12–16 deletion	Affected	Primary finding	Disease confirmed
DP10	2014	Positive parental carrier screening	Fetal sex determination	*F8*	XLR	Familial F8 variant	No further molecular testing	Not included in gene-level analysis	Female fetus; no further molecular testing
DP11	2015	Familial X-linked risk	Fetal sex determination	*DMD*	XLR	Familial DMD variant not characterized	No further molecular testing	Not included in gene-level analysis	Female fetus; no further molecular testing
DP12	2015	Previously affected child or family member	MLPA	*SMN1*	AR	SMN1 exons 7–8 deletion, heterozygous	Unaffected carrier	Primary finding	Carrier status only; disease not expected

Study ID	Year	Clinical indication	Diagnostic strategy	Gene	Mode of inheritance	Variant	Fetal molecular status	Relationship to primary indication	Final clinical interpretation
DP13	2017	Previously affected child or family member	Targeted familial variant testing by Sanger sequencing	*COL7A1*	AR	c.2305_2314delinsTT (p.Val769Phefs*3)	Unaffected carrier	Primary finding	Carrier status only; disease not expected
DP14[b]	2017	Previously affected child or family member	Fetal sex determination	*DMD*	XLR	Familial DMD exons 45–52 duplication	No further molecular testing	Not included in gene-level analysis	Female fetus; no further molecular testing
DP15	2018	Positive parental carrier screening	Targeted familial variant testing by Sanger sequencing	*SMPD1*	AR	c.1268A>G (p.His423Arg) + c.1685T>A (p.Met562Lys)	Affected	Primary finding	Disease confirmed
DP16	2018	Fetal ultrasound abnormality	Whole-exome sequencing	*CFTR*	AR	c.2991G>C (p.Leu997Phe) + c.3454G>C (p.Asp1152His)	Affected	Primary finding	Disease confirmed
DP17	2018	Fetal ultrasound abnormality	Whole-exome sequencing	*PKHD1*	AR	c.107C>T (p.Thr36Met) + c.11439C>G (p.Phe3813Leu)	Affected	Primary finding	Disease confirmed
DP18[b]	2018	Positive parental carrier screening	Fetal sex determination	*DMD*	XLR	Familial DMD exons 45–52 duplication	No further molecular testing	Not included in gene-level analysis	Female fetus; no further molecular testing
DP19	2018	Affected parent with a known autosomal dominant condition	Targeted familial variant testing by Sanger sequencing	*COL1A1*	AD	c.1155+1G>C	Unaffected	Primary finding	Familial variant excluded
DP20	2018	Fetal ultrasound abnormality	Single-gene NGS panel	*CFTR*	AR	TG10-T7-1540A + TG10-T9-1540G	Unaffected	Primary finding	No pathogenic CFTR variant associated with classic or moderate cystic fibrosis identified; detected intronic haplotypes were not considered diagnostic.
DP21	2019	Fetal ultrasound abnormality	Single-gene NGS panel	*CFTR*	AR	TG10-T7-1540A, homozygous	Unaffected	Primary finding	No pathogenic CFTR variant identified; detected 7T/7T haplotype is not associated with cystic fibrosis.
DP22	2019	Fetal ultrasound abnormality	Single-gene NGS panel	*CFTR*	AR	IVS8(TG)n-(T)n: TG[10]T[7]	Unaffected	Primary finding	No pathogenic CFTR variant identified; detected intronic haplotype is not

Study ID	Year	Clinical indication	Diagnostic strategy	Gene	Mode of inheritance	Variant	Fetal molecular status	Relationship to primary indication	Final clinical interpretation
									associated with cystic fibrosis.
RF02	2019	Postnatal index case	Whole-exome sequencing	*CHD7*	AD	c.4379_4380del (p.Ile1460Argfs*15)	Not applicable	Familial-context reference	De novo variant in postnatal index case; retained for familial molecular context
DP23	2019	Previously affected child or family member	Targeted familial variant testing by Sanger sequencing	*COL7A1*	AR	c.3140-2A>G	Unaffected non-carrier	Primary finding	Familial variant excluded
DP24	2021	Positive parental carrier screening	Targeted familial variant testing by Sanger sequencing	*GJB2*	AR	c.35del (p.Gly12ValfsTer2)	Unaffected carrier	Primary finding	Carrier status only; disease not expected
DP25	2021	Previously affected child or family member	Targeted familial variant testing by Sanger sequencing	*LIFR*	AR	c.1418del (p.Ser473Leufs*15)	Unaffected carrier	Primary finding	Carrier status only; disease not expected
DP26	2021	Positive parental carrier screening	Targeted familial variant testing by Sanger sequencing	*DHCR7*	AR	c.452G>A (p.Trp151*)	Unaffected carrier	Primary finding	Carrier status only; disease not expected
DP27	2021	Fetal ultrasound abnormality	Single-gene NGS panel	*CFTR*	AR	IVS8 Tn: 7T/7T	Unaffected non-carrier	Primary finding	No pathogenic CFTR variant identified
DP28[c]	2022	Positive parental carrier screening	Multi-gene NGS panel	*CFTR*	AR	c.1210-34TG[11]T[5]	Unaffected	Additional finding	Familial variant excluded
				HFE	AR	c.187C>G (p.His63Asp), heterozygous	Unaffected carrier	Primary finding	Carrier status only; disease not expected
RF03	2022	Postnatal index case	Whole-exome sequencing	*TRNT1*	AR	c.428_431del (p.Asp143Glyfs*12) + c.1246A>G (p.Lys416Glu)	Not applicable	Familial-context reference	Postnatal index case; retained for familial molecular context
DP29	2023	Previously affected child or family member	Whole-exome sequencing	*TUBA1A*	AD	c.1205G>A (p.Arg402His)	Unaffected	Primary finding	Familial variant excluded
DP30[c]	2023	Positive parental carrier screening	Multi-gene NGS panel	*CFTR*	AR	c.1210-34TG[11]T[5], homozygous	Homozygous	Primary finding	Homozygous low-penetrance finding; not equivalent to a classic CF-causing genotype
				HFE	AR	c.187C>G (p.His63Asp), heterozygous	Unaffected carrier	Additional finding	Carrier status only; disease not expected
DP31	2024	Fetal ultrasound abnormality	Single-gene NGS panel	*CFTR*	AR	c.1210-12T[7]/c.1210-12T[9] (IVS8 7T/9T)	Unaffected non-carrier	Primary finding	No pathogenic CFTR variant identified
DP32[d]	2024		Whole-exome sequencing	*TRNT1*	AR	c.428_431del (p.Asp143Glyfs*12), mat	Unaffected carrier	Primary finding	Carrier status only; disease not expected

Study ID	Year	Clinical indication	Diagnostic strategy	Gene	Mode of inheritance	Variant	Fetal molecular status	Relationship to primary indication	Final clinical interpretation
		Previously affected child or family member		*SERPINC1*	AD	c.391C>T (p.Leu131Phe)	Affected	Primary finding	Disease confirmed
				C7	AR	c.633_643del (p.Ser212Hisfs*4)	Unaffected non-carrier	Primary finding	Familial variant excluded
				TBX1	AD	c.248C[1] (p.Ala84Profs*18) + other 14 VUS	Heterozygous (VUS)	Additional finding	Uncertain significance; not a confirmed molecular diagnosis
				CAPN3	AD/AR (dual pattern)	c.550del (p.Thr184ArgfsTer36), heterozygous	Heterozygous	Additional finding	Incidental finding; not explanatory for the primary indication
DP33	2024	Previously affected child or family member	Single-gene NGS panel	*CFTR*	AR	c.3909C>G (p.Asn1303Lys) + c.1521_1523del (p.Phe508del), not detected in fetus	Unaffected non-carrier	Primary finding	Familial variants excluded
DP34	2024	Fetal ultrasound abnormality	Whole-exome sequencing	*CENPJ*	AR	c.3216+14C>G + c.3216+6C>G	Compound heterozygous (VUS)	Primary finding	Uncertain significance; main clinical suspicion in the source report
				RNPC3	AR	c.357_358delAA (p.Arg120Valfs*2) + c.638T>C (p.Met213Thr)	Compound heterozygous (VUS)	Additional finding	Possible contributor to the phenotype; not independently confirmed
				MYH7	AD	c.5203T>A (p.Ser1735Thr)	Heterozygous (VUS)	Additional finding	Uncertain significance; not explanatory for the primary indication
DP35	2025	Previously affected child or family member	Whole-exome sequencing	*HPRT1*	XLR	c.597delC (p.Phe199LeufsTer4)	Unaffected non-carrier	Primary finding	Familial variant excluded
DP36[d]	2025	Previously affected child or family member	Whole-exome sequencing	*C7*	AR	c.633_643del (p.Ser212Hisfs*4)	Unaffected carrier	Primary finding	Carrier status only; disease not expected
				TRNT1	AR	c.428_431del + c.1246A>G, not detected in fetus	Unaffected non-carrier	Primary finding	Familial variant excluded
				SERPINC1	AD	c.391C>T (p.Leu131Phe)	Unaffected	Additional finding	Familial variant excluded
				CAPN3	AD/AR (dual pattern)	c.550del (p.Thr184ArgfsTer36), heterozygous	Heterozygous	Additional finding	Incidental finding; not explanatory for the primary indication
DP37	2025	Affected parent with a known autosomal	Whole-exome sequencing	*NF1*	AD	c.3916C>T (p.Arg1306Ter)	Unaffected	Primary finding	Familial variant excluded

Study ID	Year	Clinical indication	Diagnostic strategy	Gene	Mode of inheritance	Variant	Fetal molecular status	Relationship to primary indication	Final clinical interpretation
		dominant condition							
DP38	2025	Positive parental carrier screening	Whole-exome sequencing	*CFTR*	AR	c.1521_1523del (p.Phe508del) + c.1251C>A (p.Asn417Lys)	Unaffected carrier	Primary finding	Carrier status only; second CFTR variant not considered clinically significant
DP39	2026	Previously affected child or family member	Whole-exome sequencing	*TUBA1A*	AD	c.425_428delinsGCTC (p.Asn142_Leu143delinsSerSer)	Heterozygous (VUS++)	Primary finding	Uncertain significance; considered jointly with GJB2 in the source report
				GJB2	AR	c.35del (p.Gly12ValfsTer2) + c.269T>C (p.Leu90Pro)	Affected	Primary finding	Considered jointly with TUBA1A in the source report
DP40	2026	Fetal ultrasound abnormality	Whole-exome sequencing	*TAB2*	AD	c.1939+1370G>A, heterozygous + c.6+60618_6+60619inv, homozygous	Affected	Primary finding	TAB2 was considered the main candidate for the cardiac phenotype; both variants were classified as VUS by the laboratory and the molecular diagnosis was not formally confirmed
				DNAH7	AR	c.11947C>T (p.Arg3983Trp), heterozygous	Heterozygous (VUS)	Additional finding	Uncertain significance; not explanatory for the primary indication
				GJB2	AR	c.35del (p.Gly12ValfsTer2) + c.457G>A (p.Val153Ile)	Unaffected carrier	Additional finding	Carrier status only; second GJB2 variant not considered clinically significant

Note: Study IDs were assigned sequentially within the complete diagnostic pathway and do not correspond to the original clinical record numbers. Pregnancies from the same family are indicated by matching superscript lowercase letters ([a,b,c,d]). Multiple gene-level findings identified within the same pregnancy are presented on separate rows under a single merged Study ID. Pregnancies investigated solely by fetal sex determination are retained in the table for cohort completeness but are classified as "Not included in gene-level analysis" and do not contribute to the gene-spectrum analysis or Figure 4B.

Abbreviations: DP, diagnostic-pathway pregnancy identifier; RF, reference familial record; mat, maternally inherited; pat, paternally inherited; AD, autosomal dominant; AR, autosomal recessive; XLR, X-linked recessive; VUS, variant of uncertain significance; MLPA, multiplex ligation-dependent probe amplification; NGS, next-generation sequencing; WES, whole-exome sequencing; PCR, polymerase chain reaction.
